# An International, Multicenter Retrospective Observational Study to Assess Technical Success and Clinical Outcomes of Patients Treated with an Endovascular Aneurysm Sealing Device for Type III Endoleak

**DOI:** 10.1177/15266028211031933

**Published:** 2021-08-03

**Authors:** Aleksandra C. Zoethout, Shirley Ketting, Clark J. Zeebregts, Dimitri Apostolou, Barend M.E. Mees, Patrick Berg, Hazem El Beyrouti, Jean-Paul P.M. De Vries, Francesco Torella, Mattia Migliari, Roberto Silingardi, Michel M.P.J. Reijnen

**Affiliations:** 1 Department of Surgery, Division of Vascular Surgery, University Medical Center Groningen, University of Groningen, Groningen, The Netherlands; 2 Department of Vascular Surgery, Rijnstate, Arnhem, The Netherlands; 3 Department of Vascular Surgery, Maastricht University Medical Center and CARIM School for Cardiovascular Diseases, Maastricht University, Maastricht, The Netherlands; 4 Department of Vascular Surgery, Santa Croce e Carle General Hospital, Cuneo, Italy; 5 Department of Vascular & Endovascular Surgery, Katholisches Karl-Leisner-Klinikum, Marienhospital Kevelaer, Kevelaer, Germany; 6 Department of Cardiothoracic and Vascular surgery, Johannes Gutenberg University Medical Center, Mainz, Germany; 7 Department of Vascular Surgery, St Antonius Hospital, Nieuwegein, The Netherlands; 8 Liverpool Vascular and Endovascular Service, Liverpool University Hospitals NHS Trust, Liverpool, UK; 9 Department of Vascular Surgery, Azienda Ospedaliero-Universitaria di Modena, University of Modena and Reggio Emilia, Modena, Italy; 10 Multimodality-Medical Imaging Group, University of Twente, Enschede, The Netherlands

**Keywords:** endovascular aneurysm repair, reintervention, abdominal aortic aneurysm, endoleak, aneurysm

## Abstract

**Introduction:**

Type III endoleaks post-endovascular aortic aneurysm repair (EVAR) warrant treatment because they increase pressure within the aneurysm sac leading to increased rupture risk. The treatment may be difficult with regular endovascular devices. Endovascular aneurysm sealing (EVAS) might provide a treatment option for type III endoleaks, especially if located near the flow divider. This study aims to analyze clinical outcomes of EVAS for type III endoleaks after EVAR.

**Methods:**

This is an international, retrospective, observational cohort study including data from 8 European institutions.

**Results:**

A total of 20 patients were identified of which 80% had a type IIIb endoleak and the remainder (20%) a type IIIa endoleak. The median time between EVAR and EVAS was 49.5 months (28.5–89). Mean AAA diameter prior to EVAS revision was 76.6±19.9 mm. Technical success was achieved in 95%, 1 patient had technical failure due to a postoperative myocardial infarction resulting in death. Mean follow-up was 22.8±15.2 months. During follow-up 1 patient had a type Ia endoleak, and 1 patient had a new type IIIa endoleak at an untreated location. There were 5 patients with aneurysm growth. Five patients underwent AAA-related reinterventions indications being: growth with type II endoleak (n=3), type Ia endoleak (n=1), and iliac aneurysm (n=1). At 1-year follow-up, the freedom from clinical failure was 77.5%, freedom from all-cause mortality 94.7%, freedom from aneurysm-related mortality 95%, and freedom from aneurysm-related reinterventions 93.8%.

**Conclusion:**

The EVAS relining can be safely performed to treat type III endoleaks with an acceptable technical success rate, a low 30-day mortality rate and no secondary ruptures at short-term follow-up. The relatively low clinical success rates, related to reinterventions and AAA enlargement, highlight the need for prolonged follow-up.

## Introduction

Endovascular aneurysm repair (EVAR) has surpassed open surgical repair for infrarenal abdominal aortic aneurysms (AAA), related to its lower morbidity and an early survival benefit.^1^ However, effective solutions must be found for late complications after EVAR. Endograft designs have evolved^2^ but complications continue to occur and warrant long-term follow-up and effective treatment.

Type III endoleak is defined as leakage between different parts of an endograft.^3,4^ This can be either due to modular disconnection (type IIIa) or due to endograft fabric disruption (type IIIb).^5,6^ Once detected, these endoleaks warrant treatment because they lead to increased pressure within the aneurysm sac, which in turn leads to an increased risk of rupture.^7^ Maleux et al^8^ reported an incidence of type III endoleak of 2.1% within 4 years after EVAR, of which 56% were type IIIa and 44% type IIIb. Endovascular treatment of type IIIa endoleak is generally performed by means of endograft relining, reconnecting the divided components of the endograft. The treatment of type IIIb endoleak is often less straightforward because the graft defect is regularly located in the area of the flow divider. As a consequence, the use of a cuff extension may not seal the tear. Relining with a bifurcated EVAR device is also often not possible because as distance from the renal artery to the flow-splitter of the original graft is often too short to host the length of a new device and its contralateral limb. A custom-made device with an inverted limb is a valid alternative, but not always available and costly. An aorto-uni-iliac device is therefore often used but requires an extra-anatomical femoro-femoral bypass, with its own morbidity. A recurrence of type III endoleak has been reported in up to 25% of patients after relining.^8^ Alternatively, open surgical conversion remains an option.^5^

Endovascular aneurysm sealing (EVAS), using the Nellix Endovascular Aneurysm Sealing system (Endologix, Irvine, CA, USA) might provide a novel and more straightforward treatment option for type III endoleaks. This can be particularly valuable in case of type IIIb endoleaks with a defect near the flow divider. With the use of EVAS, the endobags may completely fill the endograft and seal the tear. In case of a type IIIa endoleak, this could resolve the modular disconnection and might additionally provide stability. EVAS has already been successfully applied in type Ia endoleaks after prior EVAR or EVAS on short-term follow-up.^9-13^

So far only a few cases^14-18^ have been described to present the outcome of EVAS for type III endoleaks, showing technical feasibility. The objective of this study was to analyze clinical outcomes of EVAS for type III endoleaks after EVAR on a larger scale.

## Methods

### Study Design

This is an international, multicenter, retrospective observational study. A request for participation in this study was sent to centers having experience with EVAS. Local medical ethical approval and patient consent was arranged at each site in accordance with local rules and regulations prior to submission of cases. Each participating center completed a case record form (CRF) for each patient, based on hospital records and imaging. Local medical ethical guidelines were adhered to at each site according to national and local guidelines prior to data collection. Personal data was anonymized and handled in compliance with the Dutch Personal Data Protection ACT (in Dutch: Wet Bescherming Persoonsgegevens, WBP). The study was conducted according to the principles of the Declaration of Helsinki (64th WMA General Assembly, Fortaleza, Brazil, 2013) and in accordance with the applicable guidelines, regulations, and acts.

In order to be eligible, a patient had to have a history of an EVAR procedure, which was complicated by a type IIIa or type IIIb endoleak that was subsequently treated with EVAS. No exclusion criteria were applicable.

### Study Procedure

Details of a regular EVAS procedure have been described in previous publications.^1,19^ The deployment of a Nellix Endovascular Aneurysm Sealing system within the lumen of an earlier placed endoprosthesis is comparable to a primary EVAS procedure. All centers performed the procedure based on their experience with EVAS. After bilateral femoral access the EVAS systems were positioned in the desired location within the lumen of the EVAR device, fully covering the type III endoleak with sufficient proximal and distal sealing length. Subsequently the endobags were filled with polymer, with or without a prior pre-fill with saline solution. If the seal was insufficient, there was the possibility to perform a secondary fill, as in regular EVAS cases.

### Endpoints

The primary study endpoint was technical success. Other endpoints included the freedom from reintervention for the resolution of any type I or III endoleak, device occlusion and device migration ≥5 mm within 1 year after EVAS and all clinical outcomes of this patient population during the entire available follow-up. This included survival, AAA-related death, the occurrence of endoleak, device stenosis or occlusion, device migration ≥5 mm, AAA growth, and graft infection.

The endpoints were defined according to the reporting standards of the Society for Vascular Surgery.^20^ Technical success was defined as a successful introduction and deployment of the device without conversion, death, type I or III endoleak, or graft limb occlusion within 24 hours after the procedure. AAA growth was defined as 10 mm or more increase in maximal AAA diameter compared to the diameter at the first CT after the EVAS procedure. AAA-related mortality was defined as death by AAA rupture, the consequences of a primary or secondary procedure, or a surgical conversion. Clinical success was defined as successful deployment of the endovascular device at the intended location without death as a result of aneurysm-related treatment, type I or III endoleak, graft migration, graft infection or thrombosis, AAA expansion of 5 mm or more, AAA rupture, or conversion to open repair.

Comorbidities were scored according to the Society for Vascular Surgery (SVS) comorbidity grading scale.^21^ The patients were subdivided into groups for American Association for Anesthesiologists (ASA) grade 2 and ASA grade ≥2. Hypertension was defined as known history of hypertension or use of antihypertensive medication. Hyperlipidemia was defined as known history or the use of a statin or elevated lipid levels (low-density lipoprotein, total cholesterol, and triglyceride levels above normal limits for age). A patient was considered to have diabetes mellitus (DM) when there was a history of DM or use of antidiabetic medication. Renal insufficiency was defined as a serum creatinine level of ≥2.4 mg/dL or dialysis dependency.

### Statistical Analysis

Continuous variables were presented as mean and standard deviation (SD), or as median and interquartile range (IQR) depending on distribution of the data. Distribution was determined by the Kolmogorov–Smirnov tests and observation of histograms. Categorical variables were presented as frequencies and percentages. All statistical analyses were performed using the IBM SPSS Statistics version 24.0 (IBM Corp., Armonk, NY, USA). The Kaplan–Meier survival analysis was performed with censoring for patients lost to follow-up, the graph was truncated when the standard error exceeded 10%.

## Results

### Study Cohort and Indication for Revision by EVAS

**Figure 1.**
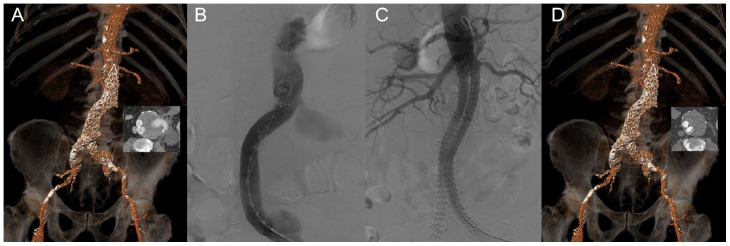
Case example of a 76 years old patient treated with endovascular aneurysm sealing (EVAS) for a ruptured 82 mm large aneurysm, due to a type IIIb endoleak of an Endurant endograft (Medtronic, Santa Rosa, CA, USA) that was inserted 7 years earlier. (A) 3D reconstruction of a pre-procedural CT scan showing the endoleak on the axial image. (B) Procedural angiography demonstrating the type IIIb endoleak. (C) Completion angiography after EVAS using bilateral Nellix Endovascular Aneurysm Sealing systems (Endologix, Irvine, CA, USA). (D) 3D reconstruction of a post-procedural CT scan showing adequate positioning of the endografts and complete exclusion of the aneurysm.

A total of 8 centers were willing to participate in the study. Overall, 20 patients who underwent secondary EVAS for type III endoleak after EVAR were included. The majority of patients had a type IIIb endoleak (n=16, 80%). One of these patients presented with an AAA rupture, related to a type IIIb endoleak (Figure 1). No other patients presented with an AAA rupture; however, 13 patients (65%) had concomitant significant AAA growth prior to revision EVAS.

### Baseline Characteristics

All but 1 patients were male. The mean age at the time of EVAR was 70.2±6.8 and 75.5±8.2 yearsat the time of EVAS. The mean BMI was 26±4.7, and the majority of patients were classified being ASA >2 (85%). The majority of patients were on medication prior to the EVAR procedure; 85% used one or more antihypertensives, 95% used antiplatelet therapy, 70% used a statin, 25% used anticoagulants, and 10% used analgesic medication. Prior to the EVAS procedure hemoglobin values were 8.2 mmol/L (IQR 6–9.2), creatinine was 96 µmol/L (IQR 80.1–122 µmol/L), and glomerular filtration rate was 60 mL/minute (IQR 46–60 mL/minute). All comorbidities are outlined in Table 1.

**Table 1.** Medical History and Comorbidities Reported in Number and Percentage.

Medical history and anatomy pre-EVAR	Number (%) or mean (SD)
Diabetes mellitus	3 (15)
Hypertension	17 (85)
Hyperlipidemia	16 (80)
Smoking (current or in past 10 years)	3 (15)
Cardiac disease	12 (60)
Renal disease	9 (45)
Pulmonary disease	10 (50)
Known peripheral artery disease	3 (15)
Prior vascular intervention	4 (20)
Thrombo-embolic event in history	2 (10)
Other concomitant aneurysm	4 (20)
Non-aneurysmal neck diameter (mm)	22.3 (1.6)
Infrarenal neck angle (degrees)	32.8 (34.4)
Infrarenal neck length (mm)	20.3 (7.1)
Maximum AAA sac diameter (mm)	59.9 (12.9)
Diameter right CIA (mm)	15 (4.1)
Diameter right femoral artery (mm)	8.6 (2.2)
Diameter left CIA (mm)	14.9 (4.1)
Diameter left femoral artery (mm)	9.1 (1.7)

Anatomy prior to EVAR procedure, reported in mean and standard deviation (SD).

Abbreviations: AAA, abdominal aortic aneurysm; CIA, common iliac artery.

### Procedural Characteristics Primary EVAR Procedure

The majority (n=15, 75%) of patients were treated with EVAR for a fusiform aneurysm. Two patients (10%) had a saccular aneurysm, 1 patient had a ruptured distal anastomotic pseudoaneurysm after previous open aneurysm repair, and in 2 cases the morphology was unknown. Anatomical details prior to the EVAR procedure are presented in Table 1.

The EVAR devices used were the AFX (Endologix, Irvine, CA, USA) (n=6, 30%), Endurant (Medtronic, Santa Rosa, CA, USA) (n=6, 30%), Cook Zenith (Cook Medical, Bloomington, IN, USA) (n=4, 20%), Talent (Medtronic, Santa Rosa, CA, USA) (n=3, 15%), and the Excluder (W.L. Gore & Associates, AZ, USA) (n=1, 5%). Two patients required adjuvant proximal stenting to improve proximal seal. One patient was treated with a Palmaz Genesis stent (Cardinal Health, Dublin, Ireland) because of a procedural type Ia endoleak, and 1 patient received a proximal cuff to improve seal without apparent type Ia endoleak. Additionally, 2 patients had adjuvant distal extensions, 1 bilaterally and 1 a left iliac extension. Further procedural details are reported in Table 2.

**Table 2.** EVAR Procedural Characteristics, Continuous Data Presented as Median and Interquartile Range (IQR), and Categorical Variables Presented as Number and Percentage.

Procedural characteristics EVAR	Median (IQR) or number (%)
Anesthesia type	
General	9 (45)
Regional	2 (10)
Local	6 (30)
Unknown	3 (15)
Access	
Cutdown	13 (65)
Percutaneous	2 (10)
Combination of cutdown and percutaneous	1 (5)
Unknown	4 (20)
Blood loss (mL)	100 (25–191.3)
Contrast volume used (mL)	75 (42.5–183.8)
Fluoroscopy time (minutes)	13 (8–20)
Procedural time (minutes)	84 (67.5–114)

Abbreviation: IQR, interquartile range.

### Procedural Characteristics EVAS Procedure

The median time between EVAR and EVAS was 49.5 months (IQR 28.5–89 months). Prior to EVAS, all patients underwent a contrast enhanced CT scan. Additionally, some patients also had duplex ultrasound examination (n=7), angiography (n=1), or a plain abdominal X-rays (n=1). The mean AAA diameter prior to EVAS revision was 76.6±19.9 mm, and all EVAS procedures were bilateral. The median used length of the Nellix device was 170 mm on both sides (IQR on right side 140–180 mm, IQR on left side 150–180 mm). All patients had antibiotic prophylaxis and most had heparin administered prior to the procedure (n=18, remaining 2 are unknown). Additional procedural characteristics can be found in Table 3.

**Table 3.** Endovascular Aneurysm Sealing (EVAS) Procedural Characteristics, Continuous Data Presented as Median and Interquartile Range (IQR), and Categorical Variables Presented as Number and Percentage.

Procedural characteristics EVAS	Median (IQR) or number (%)
Anesthesia type	
General	12 (60)
Regional	1 (5)
Local	7 (35)
Access type	
Cutdown	15 (75)
Percutaneous	5 (25)
Prefill volume (mL)	50 (40–65)
Polymer volume (mL)	44 (30–65)
Blood loss (mL)	150 (100–350)
Contrast volume used (mL)	110 (78.8–192.5)
Fluoroscopy time (minutes)	9 (8–13)
Procedural time (minutes)	108 (84–156.5)
ICU stay (days)	0 (0–1)
Postoperative hospital stay (days)	3 (2–5.5)

Abbreviation: ICU, intensive care unit.

A prefill with saline solution was performed in 15 (75%) patients, and in most of them a prefill and final fill pressure of 180 mmHg was adhered to. In 2 cases, the polymer fill pressure was more than 200 mmHg. Secondary fill was performed in 3 cases (15%). One patient died within 24 hours after EVAS due to myocardial infarction. In the remaining 19 patients, technical success was achieved (95%). There were 3 cases who had a persistent type II endoleak on the completion angiography.

### 30-Day Outcome

Besides the deceased patient, all patients had at least 30-day follow-up. No other deaths occurred within the first 30 days rendering the 30-day mortality at 5%. At 30-day follow-up, the majority of patients had contrast enhanced CT (70%), without (n=8) or with (n=6) additional imaging modalities (contrast enhanced CT with duplex ultrasound and X-ray n=5, contrast enhanced CT with duplex ultrasound n=1). Three patients only had duplex ultrasound examination, 1 patient had a CT without contrast, and 1 patient had angiography. There were 7 (35%) patients with an AAA-related complication within 30-day follow-up. Three patients (15%) had a newly formed type II endoleak not yet reported on completion angiography. In addition, 1 patient had right-sided intermittent claudication due to 60% stenosis of the right Nellix, which was treated conservatively. There were no patients with occlusion or migration of the endografts, and there were no persistent type III endoleak observed. Additionally, there were 2 patients with procedure-related complications, which included local wound infection in the groin and inguinal wound dehiscence.

### 30-Day to Latest Follow-up Outcome

The mean time to latest follow-up was 22.8±15.2 months. Between 30 days and latest follow-up, there were 3 patients who had a newly reported endoleak; 1 type II at 1 month, 1 type Ia at 5 months, and 1 type IIIa at 33 months, all without AAA growth. The patient with the type II endoleak was treated conservatively, but the 2 others underwent a reintervention. The patient with a type Ia endoleak had a concomitant symptomatic AAA and due to the semi-urgent setting and poor general condition of the patient, there was insufficient time to perform a fenestrated cuff or chimney procedure. A bare-metal cuff was placed to improve apposition of the grafts with a successful outcome. The patient with a type IIIa endoleak, previously treated for a type IIIb endoleak had a disconnection of the EVAR device below the location of the Nellix EVAS system and underwent relining with another endograft. In this case, the chosen Nellix EVAS legs used in the EVAS relining procedure were too short to cover the connection site between legs and body and were not extended because the reason for reintervention was a type IIIb endoleak.

There were 4 patients, already diagnosed with a type II endoleak within 30 days or directly postoperatively, that showed significant AAA growth during later follow-up. They had growth of 36, 35, 13, and 12 mm at 27, 25, 12, and 42 months, respectively. The patient with a 36 mm AAA growth underwent reintervention at 26 months, a perigraft hygroma was found and evacuated, and the aneurysm was plicated and closed. This patient suffered from cardiac failure and myocardial infarction after the laparotomy and died 27 months after the initial EVAS procedure. The patient with 35 mm growth underwent explantation of the devices at 26 months after EVAS, during reintervention a type Ia endoleak was found, which had not been seen on earlier imaging. The patient with 13 mm growth underwent a laparotomy and suturing of a type II endoleak at 26 months. The patient with 12 mm aneurysm growth was treated conservatively. Additionally, there was one other patient with AAA growth without a reported endoleak and was treated conservatively to date. Another patient had a progressive iliac aneurysm distal from the EVAR device for which reintervention was performed at 16 months after EVAS. Endovascular repair was performed with an additional femoro-femoral bypass due to complete intraoperative thrombus of the left Nellix device and left iliac axis.

Eight patients died during follow-up, of which 2 were AAA-related, as described above. Additionally, 1 patient died after a surgically treated femur fracture 46 months after EVAS, 1 patient died due to heart failure 10 months after EVAS, 1 patient died of angiosarcoma of the femur 3 months after EVAS, and 1 patient died due to mesenteric ischemia 34 months after EVAS. In 2 cases, the patient died at home and no further details were available (22 and 7 months after EVAS). All complications at 30 days to latest follow-up and at 30 days are described in Table 4.

**Table 4.** Complications During Follow-Up Including Number of Complications Within First 30 Days and Number of Complications Between 30 Days and Last Follow-Up.

Complications	30 days number	30 days to latest follow-up number	Total number
Death			
Aneurysm related	1	1	2
Not aneurysm related	—	4	4
Unknown cause	—	2	2
Endoleak			
Type Ia	—	1	1
Postoperative type II	3	—	3
Newly formed type II	3	1	4
Type III	—	1	1
Aneurysm growth	na	5	5
Stenosis or occlusion of the Nellix device	1	1	2
AAA-related reintervention	—	5	5
Other procedure related	2	—	2

Abbreviation: AAA, abdominal aortic aneurysm.

### Survival Analysis

**Figure 2.**
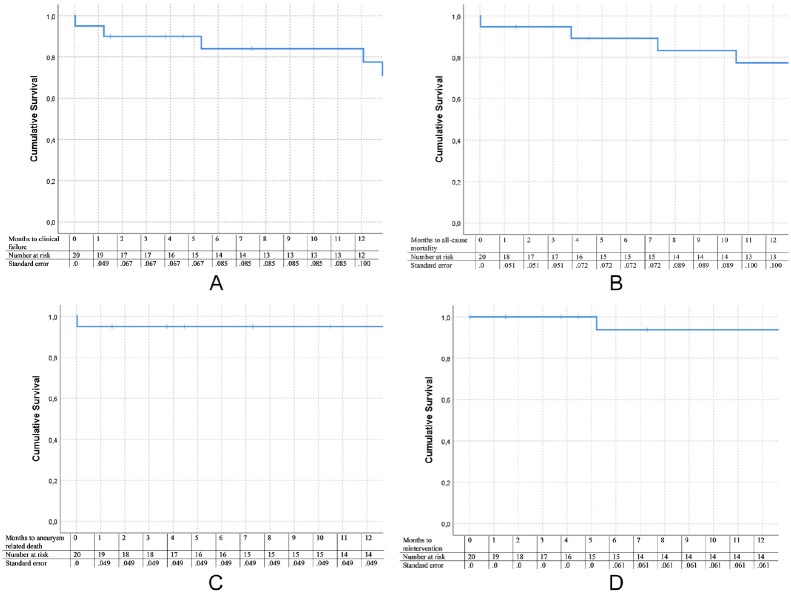
Survival curves performed with Kaplan–Meier analysis. (A) Freedom from clinical failure. (B) Freedom from all-cause mortality. (C) Freedom from aneurysm-related mortality. (D) Freedom from reintervention.

All Kaplan–Meier curves are depicted in Figure 2. Freedom from clinical failure was 95% at 30 days and 77.5% at 1 year. Freedom from all-cause mortality was 94.7% at 30 days and 77.3% at 1 year, with a freedom from AAA-related mortality of 95% at both 30 days and 1 year. Finally, freedom from AAA-related reinterventions was 100% at 30 days and 93.8% at 1 year. This includes only AAA-related reinterventions and does not include the access site-related reinterventions or reinterventions for more distal thrombosis or occlusion.

## Discussion

The current study gives a representation of the results of EVAS performed as a relining procedure for type III endoleak after EVAR. Even though this is a procedure that is outside of the current instructions for use of EVAS, a technical success rate of 95% is observed without secondary AAA ruptures. The results show that this technique can be used effectively to treat a type III endoleak after EVAR, although a significant number of patients still require additional reinterventions, emphasizing the need for long-term follow-up. However, it must be noted that re-reintervention after EVAS-relining can be challenging, because the proximal aorta is less accessible after EVAS. This was made eminent by the case of a type Ia endoleak, which emphasizes the need for appropriate diagnostic screening prior to EVAS relining.

There were 2 AAA-related deaths. One occurred within 24 hours after EVAS and the other after a secondary reintervention, both having a cardiac cause. This stresses the frailty of the population and highlights the importance of a proper patient selection. Vascular patients are known to have (cardiovascular) comorbidities, as has been reported in our cohort with 60% of cases having cardiac diagnosis prior to EVAR and 85% of patients with hypertension and caution must be operated when performing (re)interventions. Importantly, the patient who died after reintervention had a suspected type IIIb endoleak which had never been demonstrated, on the reintervention a perigraft hygroma was found. As such, it is possible that this patient in fact never had a type IIIb endoleak but that the hygroma was misdiagnosed for an endoleak.

Compared to other reinterventions, performed for type III endoleak after EVAR, the use of EVAS for relining seems to be a valuable alternative. Skibba et al^22^ described 17 cases with a type IIIa endoleak after EVAR who underwent EVAR-relining of which three patients presented with a recurrent type IIIa endoleak and a ruptured AAA. In our cohort there were no AAA ruptures and only 1 recurrent type III endoleak. This patient presented with a new type IIIa endoleak, below the Nellix stents, after a type IIIb endoleak with aneurysm growth had been treated with EVAS and was consequently not related to the EVAS procedure itself. However, in retrospect, the cause of aneurysm growth in this case might have been the type IIIa endoleak, which was not recognized as such at the time. The preferred treatment at the time would have been re-relining with EVAS; however, this was not possible since at this time the EVAS system had been taken of the market, which complicated matters for this case. Prior to this study, several cases of EVAS for the treatment of a type III endoleak have been described. Van der Ham et al^14^ described 2 cases of which 1 patient died 7 months after the procedure unrelated to the AAA, and 1 patient had an uneventful follow-up of 6 months. Additionally, Lareyre et al^15^ described a cohort of 10 cases which included 3 patients with a type III endoleak, treated with EVAS. Two complications were seen: a reintervention for a type II endoleak and for a type Ia endoleak, but it remained unclear whether these complications were in the group of patients treated for a type III endoleak. A number of other studies have been performed reviewing EVAS for failed previous EVAR procedures^16,17^ on a small number of patients (n=4, n=5 type III endoleak), which showed the technique to be feasible, and outcomes were promising on the short term. As such, this is the largest study to date to report the outcomes of EVAS for type III endoleak with the longest follow-up. From the limited studies yet performed, it seems that the results from our study fall in line with previous case reports.

Of all observed complications, type II endoleak was the most prevalent and occurred in 7 patients. Six of them were observed at 30-day follow-up or directly postoperative. Due to the design of EVAS, type II endoleak after EVAS alone is highly unlikely and has been reported to be as low as 0.6%.^17^ Due to positioning of the Nellix stents inside an EVAR device in this technique, coexisting type II endoleaks are not treated. As such, the most likely scenario is that the type II endoleak was present prior to EVAS relining and was not truly a complication of the performed reintervention. In our cohort, there were 5 patients that had AAA growth, regardless of a successful treatment of the type III endoleak. Four of them were related to a persistent type II endoleak. From the ENGAGE registry, it was recently shown that type II endoleaks are related to an unfavorable AAA sac remodeling with more AAA growth.^23^ The side branches may have served as an outflow for the type III endoleak but after treatment the flow might be reversed, leading to a type II endoleak. Some cases have been described in literature of combinations of type II and type III endoleaks.^24^

From a technical point of view, the placement of a Nellix stent inside an EVAR device does not significantly differ from a regular EVAR. However, the required volume of polymer is lower when compared regular EVAS. Lower volumes are related to a steeper increase in the pressure in the endobags. Therefore, the polymer should be injected at a slow speed. In the current study, the final pressures were in line with those advised for regular EVAS. Prefill was only performed in 75% of cases, but might be of utmost important to unwrinkled the endobags before polymer injection, particularly when low volumes are used.

The clinical success was relatively low at 77.5% at 1 year but must be interpreted with caution due to small numbers. The main causes for clinical failure were the reinterventions and AAA growth. Despite the likeliness that AAA growth was related to type II endoleak this is an important predictor of aneurysm rupture and death and should not be taken lightly. Low clinical success rates at 1 year might be a predictor for further clinical failures and warrants the need of thorough follow-up for these patients.

Some limitations to this study exist. The data were collected from different centers and heterogeneity might be expected due to this. The participating centers based their decision to use EVAS relining on the most recent research and knowledge, but the decision process might differ across centers. Since the data collection was retrospective, our study will not have influenced the decision-making of the centers. Additionally, it is likely that differences in experience have led to varying outcomes and that diagnostic and treatment modalities vary slightly at each center. Additionally, even though this is the largest study evaluating the results of EVAS for type III endoleak, the sample size is still small with only 20 patients of which only 10 had 2 year follow-up or more. As such, this must be taken into account when interpreting the data. It must be acknowledged that the use of Nellix as relining is outside of the instructions for use (IFU) of the device, and this carries a greater risk of complications and reintervention. However, on occasions where no ideal reintervention exists for complications such as type III endoleak, creativity is crucial in order to find more or less suitable solutions. Since after both EVAR and EVAS, most complications generally occur at later follow-up stages a study with longer follow-up would be valuable.

Finally, as mentioned earlier, the Nellix device is currently not available for clinical use. In January 2019, unforeseen complications led to the stop of unrestricted sales and commercial use of the device. The main reason for this being a higher than anticipated migration and endoleak rate^25^ leading to complex open conversions. However, sac-sealing devices might still have a future as primary procedures or as adjuncts for reintervention in complications, such as type III endoleak. Additionally, it has been announced that a CE Mark certification for the Nellix device has been reinstated after an assessment of clinical evidence.^26^ It will be interesting to see which adjustments are made to the device and how our current knowledge of the device will aid us in treating patients better in the future.

## Conclusion

This study showed that EVAS relining can be safely performed to treat type III endoleaks with an acceptable technical success rate, a low 30-day mortality rate, and no secondary ruptures. Compared to relining by EVAR this is a valuable alternative. However, a high AAA-related mortality emphasizes the importance of thorough preoperative screening and endoleak classification. Additionally, the low clinical success rates, related to reinterventions and AAA enlargement, highlight the need for prolonged follow-up.
